# Fine mapping of a novel QTL *DM9.1* conferring downy mildew resistance in melon

**DOI:** 10.3389/fpls.2023.1202775

**Published:** 2023-06-12

**Authors:** Xuejun Zhang, Yueming Ling, Wenli Yang, Minghua Wei, Zhenzhu Wang, Meihua Li, Yong Yang, Bin Liu, Hongping Yi, Yang-Dong Guo, Qiusheng Kong

**Affiliations:** ^1^ College of Horticulture, China Agricultural University, Beijing, China; ^2^ Hami-melon Research Center, Xinjiang Academy of Agricultural Sciences, Urumqi, China; ^3^ Hainan Sanya Experimental Center for Crop Breeding, Xinjiang Academy of Agricultural Sciences, Sanya, China; ^4^ College of Horticulture and Forestry Sciences, Huazhong Agricultural University, Wuhan, China

**Keywords:** QTL-seq, QTL, melon, linkage map, downy mildew

## Abstract

Downy mildew (DM) is a major foliar disease globally causing great economic loss in melon production. Utilizing disease-resistant cultivars is the most efficient approach for disease control, while discovery of disease-resistant genes is crucial for the success of DM-resistant breeding. To address this problem, two F_2_ populations were constructed using the DM-resistant accession PI 442177 in this study, and QTLs conferring DM resistance were mapped using linkage map and QTL-seq analysis, respectively. A high-density genetic map with the length of 1096.7 cM and density of 0.7 cM was generated by using the genotyping-by-sequencing data of a F_2_ population. A major QTL *DM9.1* with the phenotypic variance explained proportion of 24.3-37.7% was consistently detected at the early, middle, and late growth stages using the genetic map. QTL-seq analyses on the two F_2_ populations also validated the presence of *DM9.1*. Kompetitive Allele-Specific PCR (KASP) assay was further carried out to fine map *DM9.1* into 1.0 Mb interval. A KASP marker co-segregating with *DM9.1* was successfully developed. These results not only provided valuable information for DM-resistant gene cloning, but also offered useful markers for melon DM-resistant breeding programs.

## Introduction

Melon (*Cucumis melo* L.) is an economically important horticultural crop and extensively cultivated for its fleshy fruits. According to the statistics of FAOSTAT, the global harvested area of melon was 1,068,238 ha in 2020, and the top five producing nations were China, Türkiye, India, Afghanistan, and Iran (FAOSTAT, 2022). However, in many planting areas, diseases are the major threats for melon production.

Downy mildew (DM), incited by the obligate oomycete pathogen *Pseudoperonospora cubensis*, is the most devastating foliar disease not only for melon but also for most of the other species in Cucurbitaceae family (Rahman et al., 2021). Foliar chlorosis and necrosis are the typical symptoms of DM, which can occur at all developmental stages and decrease the yield and fruit quality of melon dramatically. This disease has a wide geographic distribution. DM epidemics have caused great economic losses in melon production around the world, especially in humid production areas. Currently, chemical control of DM is widely practiced in melon production. However, the chemical control probably loses its efficacy due to the prevalence of resistant strains of pathogens. The most efficient approach for DM control is to develop and use resistant cultivars. As a result, understanding the genetic basis of DM resistance and development of resistant cultivars have become a priority for the scientific community in recent years.

Several DM-resistant accessions have been identified for melon. It was reported that melon breeding line MR-1 derived from PI 124111 exhibited resistance not only to DM, but also to powdery mildew and Fusarium wilt (Cohen and Eyal, 1987). After that, 2 highly resistant, 31 resistant, and 49 moderately resistant accessions to DM were identified from 942 melon Plant Introductions (PI) by field evaluations (Thomas and Jourdain, 1992). Moreover, the other evaluation for resistance against DM identified 2 highly resistant and 68 resistant accessions from 1076 melon PIs (Thomas, 1999). These results provided valuable germplasm for melon DM-resistant breeding.

Inheritances of DM resistances were extensively investigated in melon. Genetic analysis showed that DM resistance in MR-1 was controlled by two partially dominant loci, namely *Pc-1* and *Pc-2* (Kenigsbuch and Cohen, 1989). Subsequently, *Pc-3*, *Pc-4*, and *Pc-5* were also identified in different melon accessions (Kenigsbuch and Cohen, 1992). These results suggested that different melon germplasm probably harbored different DM-resistant genes.

Genetic basis of DM resistance was dissected in several studies. A unique 45 kDa cytoplasmic soluble protein (P45) was firstly found to co-segregate with the DM resistance in melon (Balass et al., 1992). Using an unsaturated map, a major QTL for DM resistance was identified in a RIL population derived from the DM-resistant PI 124112 (Perchepied et al., 2005). The resistance of PI 124111 to DM was found to be controlled by enhanced expression of the enzymatic resistance genes *At1* and *At2* (Taler et al., 2004). The low expression of *At1* and *At2* in the susceptible melons was modulated by transcriptional inhibition (Benjamin et al., 2009). Recently, using MR-1 as the resistant parental line, 9 QTLs for DM resistance were identified and 2 of them were major QTLs locating on chromosome 8 and 10, respectively (Toporek et al., 2021).

Knowledge on the inheritance of DM resistance substantially facilitated the utilization of resistant sources in melon breeding programs. However, most of the researches focused on the resistance harbored by PI 124111. The previous studies showed that several melon accessions exhibited resistances to DM (Thomas and Jourdain, 1992; Thomas, 1999). Thus, mining and utilizing more resistant genes are crucial for melon DM-resistance breeding programs. To address this problem, two genetic populations were constructed using the DM-resistant melon accession PI 442177 in this study, and a major QTL for whole-stage DM resistance was identified at the end of chromosome 9 using both QTL-seq and linkage map-based QTL mapping approaches. The results will provide valuable information not only for DM-resistant gene cloning, but also for genetic improvement of melon with DM resistance by pyramiding the multiple resistance genes in breeding programs.

## Methods and materials

### Plant materials

Melon accession PI 442177 was reported to confer DM resistance (Thomas, 1999). In our previous experiments, it also exhibited high resistance to DM. As a result, it was selected as the DM-resistant resource. Huangtu and Huangdanzi are the landraces with desirable fruit quality but susceptible to DM. Inbred lines of the two landraces were developed through multiple rounds of self-pollination and selection. The two inbred lines were crossed with PI 442177 to produce two F_2_ populations, respectively. The F_2_ population of PI 442177 and Huangtu had 157 individuals, while, the F_2_ population of PI 442177 and Huangdanzi had 600 individuals.

### Spray inoculation and phenotyping of DM resistance

Seeds were sown into plastic trays filled with sterilized substrates and grown in a nursery greenhouse. Temperature in the nursery greenhouse ranged from 20 to 35°C. The seedling management followed the commercial production practices. The isolate of DM pathogen *P. cubensis* was recovered from the symptomatic leaf tissue of melon cultivated in Sanya, China (18°09′34″–18°37′27″ N, 108°56′30″–109°48′28″ E). The concentration of 5 × 10^3^ sporangia/mL was used for inoculation. When the first true leaf fully expanded, the seedlings were inoculated with *P. cubensis* using the spraying method. After inoculation, the seedlings were covered with a plastic tunnel and kept at 100% relative humidity to induce infection for 24 h.

For the F_2_ population derived from the parental lines of PI 442177 and Huangtu, the DM reactions were investigated at early, middle, and late growth stages, respectively. At early growth stage, the reaction levels were measured at 10^th^ day post inoculation (DPI). Then, the seedlings were transplanted into a plastic greenhouse with the spacing of 40 × 60 cm. The growth management followed the commercial production practices. At middle growth stage, DM infection levels were investigated at the 20^th^ day after transplantation. At late growth stage, the incidences of DM were investigated at the 30^th^ day after fruit setting. For the F_2_ population derived from the parental lines of PI 442177 and Huangdanzi, the incidences of DM were measured at 10^th^ and 14^th^ DPI, respectively. The DM infection levels were classified into six categories based on the ratio of symptomatic leaf area and entire leaf area: 0, absence of symptom; 1, less than 1/4; 2, 1/4-1/2; 3, 1/2-2/3; 4, 2/3-3/4; 5, larger than 3/4.

### DNA isolation

Leaves from the parental plants and F2 individuals were sampled and immediately frozen in liquid nitrogen. Genomic DNA was isolated for each sample using a Plant DNA Extraction Kit (Tiangen, China) according to its instruction. Integrity and purity of each DNA sample were measured using the 1% agarose gel electrophoresis and a NanoDrop 1000 spectrophotometer. The high-quality DNA samples were used for the subsequent sequencing.

### Genotyping-by-sequencing and variant calling

GBS was performed for Huangtu, PI 442177 and their F_2_ individuals, respectively. *HaeIII* and *RsaI* were used as the enzyme combination to construct the sequencing library according to the protocol (Elshire et al., 2011). The GBS libraries were sequenced on an Illumina HiSeq 2500 platform (Illumina, Inc.). FastQC was used to check the quality and quantity of the clean reads (https://www.bioinformatics.babraham.ac.uk/projects/fastqc/). Then, the low-quality and adaptor sequences were removed from the reads using fastp (Chen et al., 2018). An improved assembly of melon genome (v3.6.1) was used as the reference (Ruggieri et al., 2018). The clean reads were mapped to the reference genome using the BWA-MEM algorithm (Li and Durbin, 2009). The variants, including SNPs and InDels, were called and genotyped using the HaplotypeCaller of GATK4 (https://github.com/broadinstitute/gatk). The variants exhibiting homologous genotype in each parental line and polymorphism between the parental lines were selected for the downstream analysis. The effects of variants were predicted using snpEff (https://pcingola.github.io/SnpEff/).

### Genetic map construction and QTL mapping

Lep-MAP3 was used to construct the genetic map (Rastas, 2017). Briefly, the parental genotypes were firstly called using the *ParentCall2* module. Then, the loci with segregation distortion (p < 0.01) and missing genotype ratio larger than 0.3 were removed using the *ParentCall2* module. By setting the LOD scores varying from 20 to 40, the remaining SNPs were separated into linkage groups (LGs) using the *SeparateChromosomes2* module. At last, the orders of SNPs and their genetic distances were determined using the *OrderMarkers2* module. The Kosambi mapping function was used to calculate the genetic distance. The SNPs in a region without recombination were collapsed into a bin.

R/qtl2 was used for QTL analysis (Broman et al., 2019). The *scan1* function was used to perform genome scan to detect the potential QTLs using the Haley-Knott regression. One thousand permutations were performed to determine the genome-wide significance threshold for LOD (p < 0.05). QTL with the LOD exceeding the threshold value was considered significant. QTL effect and the proportion of phenotypic variance explained (PVE) by the QTL were estimated at the highest QTL peak using *fit1*. For each detected QTL, the confidence interval was defined using a 95% Bayesian credible interval. Naming of QTL followed the nomenclature recommendations in cucumber (Wang et al., 2020).

### Whole-genome re-sequencing and QTL-seq analysis

QTL-seq analysis was also used to map the DM resistance genes. In the F_2_ population derived from the parental lines of PI 442177 and Huangtu, 30 individuals showing consistent resistance to DM and 30 individuals exhibiting consistent susceptibility to DM at three growth stages were selected and their DNA samples were equally bulked to generate the resistant and susceptible pools, respectively. Similarly, in the F_2_ population of PI 442177 and Huangdanzi, 30 individuals showing consistent DM resistance and 30 individuals exhibiting consistent DM susceptibility at the two time points were selected to construct the resistant and susceptible pools, respectively. Whole-genome re-sequencing was carried out for the parents and pools on an Illumina HiSeq 2500 platform (Illumina, Inc.). Quality control and variant calling followed the pipeline of GBS data analysis. The PCR duplicates were removed before variant calling. SNP-index and delta SNP-index were calculated according to the formula described in QTL-seq analysis (Takagi et al., 2013). Distribution of delta SNP-index was plotted using the sliding window of 2 Mb and the step of 100 kb.

### Kompetitive allele-specific PCR assay

The KASP assay and the F_2_ population derived from the parental lines of Huangdanzi and PI 442177 were used to narrow down the mapping interval. The polymorphic SNP loci in the mapping interval were selected for KASP assay. Two allele-specific forward primers and one common reverse primer for the KASP assay were designed using Primer3Plus (https://www.primer3plus.com/). The PCR product size was set in the range of 60-100 bp. The GeneMatrix™ system (HC Scientific, Chengdu, China) was used for genotyping. The 2 μL PCR reaction mixture included 1 μL of 2 × KASP reaction mix, 50 ng of DNA template, 0.15 μL Hex forward primer, 0.15 μL FAM forward primer, and 0.04 μM common reverse primer. The touchdown PCR reaction was performed on a Matrix Cycler with a 10 min host-start activation step at 94°C, followed by 10 touchdown cycles of 94°C for 20 s and 61°C for 45 s (decreasing 0.3°C per cycle), then 35 cycles of 94°C for 20 s and 55°C for 20 s (decreasing 0.3°C per cycle). Fluorescence levels were measured using a Matrix Scanner. Genotypes were viewed using a Matrix Master.

## Results

### DM resistance variations in the two F_2_ populations

**Figure 1:**
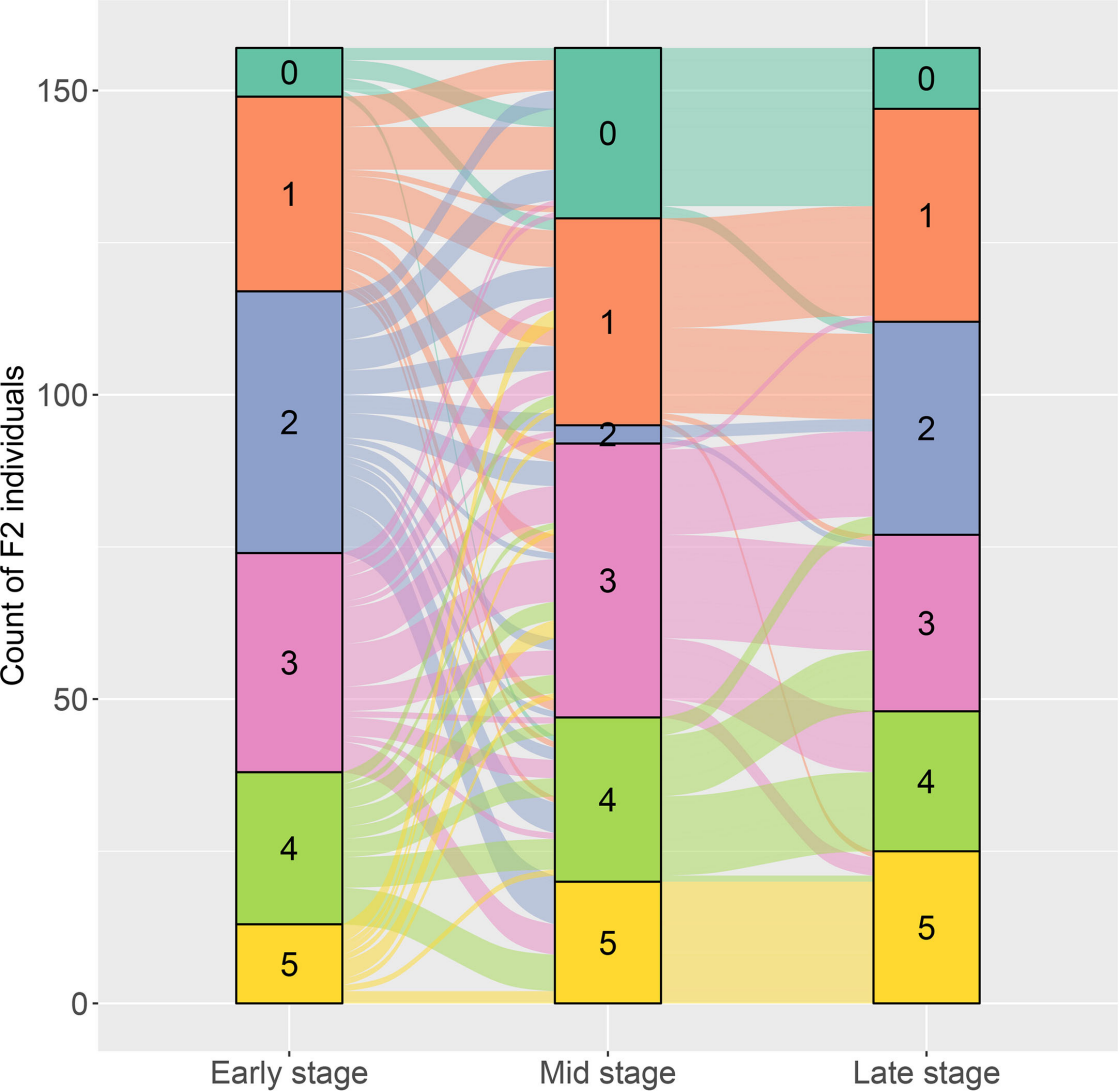
The alluvial diagram on the dynamic changes of downy mildew resistance at early, stage, and late growth stages for the F_2_ individuals derived from the cross of PI 442177 and Huangtu.

The resistant level to DM infection for each plant in the two F_2_ populations was determined according to the six grades of disease infection. For the F_2_ population of PI 442177 and Huangtu, the DM-resistant level of each plant was measured at three growth stages. Considerable phenotypic variations in response to DM infection were observed for the F_2_ individuals. The results are shown in an alluvial diagram (**Figure 1**). The resistant levels exhibited a continuous distribution at each growth stage, demonstrating that the DM resistance is a quantitative trait. Meanwhile, dynamic changes of the resistant phenotypes were observed for the same plant at different stages. Dramatic changes of the resistant phenotypes were observed between the early and middle stages for several F_2_ individuals. However, the resistant phenotypes for the same plant exhibited more stable pattern between the middle and late stages. These results indicated that DM resistance was a stage-dependent phenotype in melon, and measurement of resistant response through whole growth stage was necessary for accurately determining DM resistance of melon germplasm. Similarly, DM-resistant levels in the F_2_ population of PI 442177PI 442177 and Huangdanzi also exhibited continuous variations.

### High-density genetic map construction

GBS was performed for PI 442177, Huangtu, and their F_2_ population to construct a genetic map. A total of 57.4 Gb sequencing data were generated. The number of reads for each sample varied from 1.98 M to 6.16 M, with a mean of 3.38 M. The genome coverage ranged from 0.54 to 1.65× with an average of 0.91×. The clean reads were mapped to melon reference genome. The mapping ratios were in the range of 85.36% and 98.88% with a mean of 97.78%. GATK4 was used to identify the polymorphic loci, resulting in a total of 1,254,775 SNPs and 200,702 InDels. Only the SNP loci exhibiting homozygous genotype in each parental line and polymorphism between the parents were kept. Moreover, the loci with missing ratio larger than 0.3 were removed, resulting in 69,770 high-quality SNPs with the Ti/Tv ratio of 2.08.

**Figure 2:**
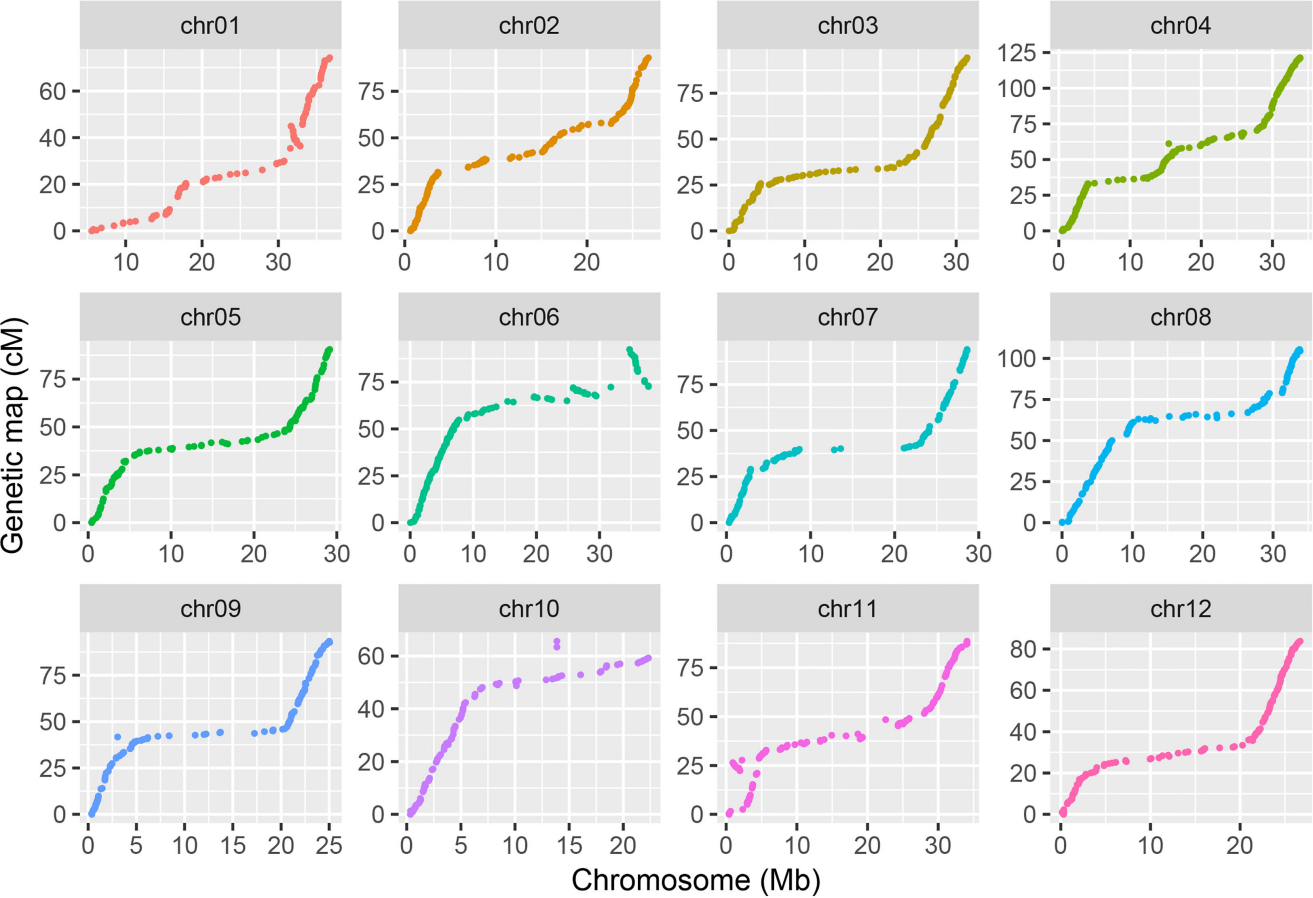
Synteny between the genetic map and reference genome.

Lep-MAP3 was used to construct the genetic map. The SNPs were further filtered using the modules of *ParentCall2* and *Filtering2* in Lep-MAP3, resulting in 347,703 SNPs. Then, the LOD scores ranging from 20 to 40 were used to separate the SNPs into LGs. At the LOD score of 28, the SNPs were grouped into the desired 12 LGs and the number of singleton was the smallest. As a result, LOD score of 28 was used to classify the SNPs into LGs. The markers were further ordered and their genetic distances were calculated in each LG. According to the physical locations of SNPs, the LGs were assigned to the corresponding chromosomes. The makers in a region without recombination event were collapsed into a bin. The first SNP in each bin was kept to represent the bin. Finally, a chromosome-level bin-map with 1,603 bins and in length of 1,096.7 cM was constructed (**Figure S1**). Summary statistics on the genetic map are listed in **Table 1**. The number of bins in each LG ranged from 96 (LG10) to 198 (LG4). The LG lengths varied from 65.6 cM (LG10) to 121.4 cM (LG4). The maximum spacing between bins varied from 3.2 cM (LG2 and LG12) to 6.4 cM (LG7). The average spacing between bins varied from 0.6 cM to 0.8 cM, with a mean of 0.7 cM. Relationships between the marker locations on the LGs and reference genome were plotted and compared. A good synteny between the genetic map and the reference genome was observed except a significant transversion event was observed at end of chromosome 6 (**Figure 2**). These results demonstrated that a high-density and chromosome-level genetic map was constructed for the F_2_ population derived from the parental lines of PI 442177 and Huangtu.

**Table 1:** Summary statistics on the genetic map.

LG/Chromosome	Number of bins	Length of LGs (cM)	Average spacing of bins (cM)	Maximum spacing of bins (cM)
1	101	74.3	0.7	5.4
2	142	93.0	0.7	3.2
3	138	94.3	0.7	6.1
4	198	121.4	0.6	4.1
5	127	90.5	0.7	3.8
6	149	92.4	0.6	4.8
7	122	94.0	0.8	6.4
8	145	105.5	0.7	4.2
9	119	93.4	0.8	4.5
10	96	65.6	0.7	4.1
11	133	88.6	0.7	5.1
12	133	83.8	0.6	3.2
Overall	1603	1096.7	0.7	6.4

### QTL mapping of DM resistance based on the genetic map

**Figure 3:**
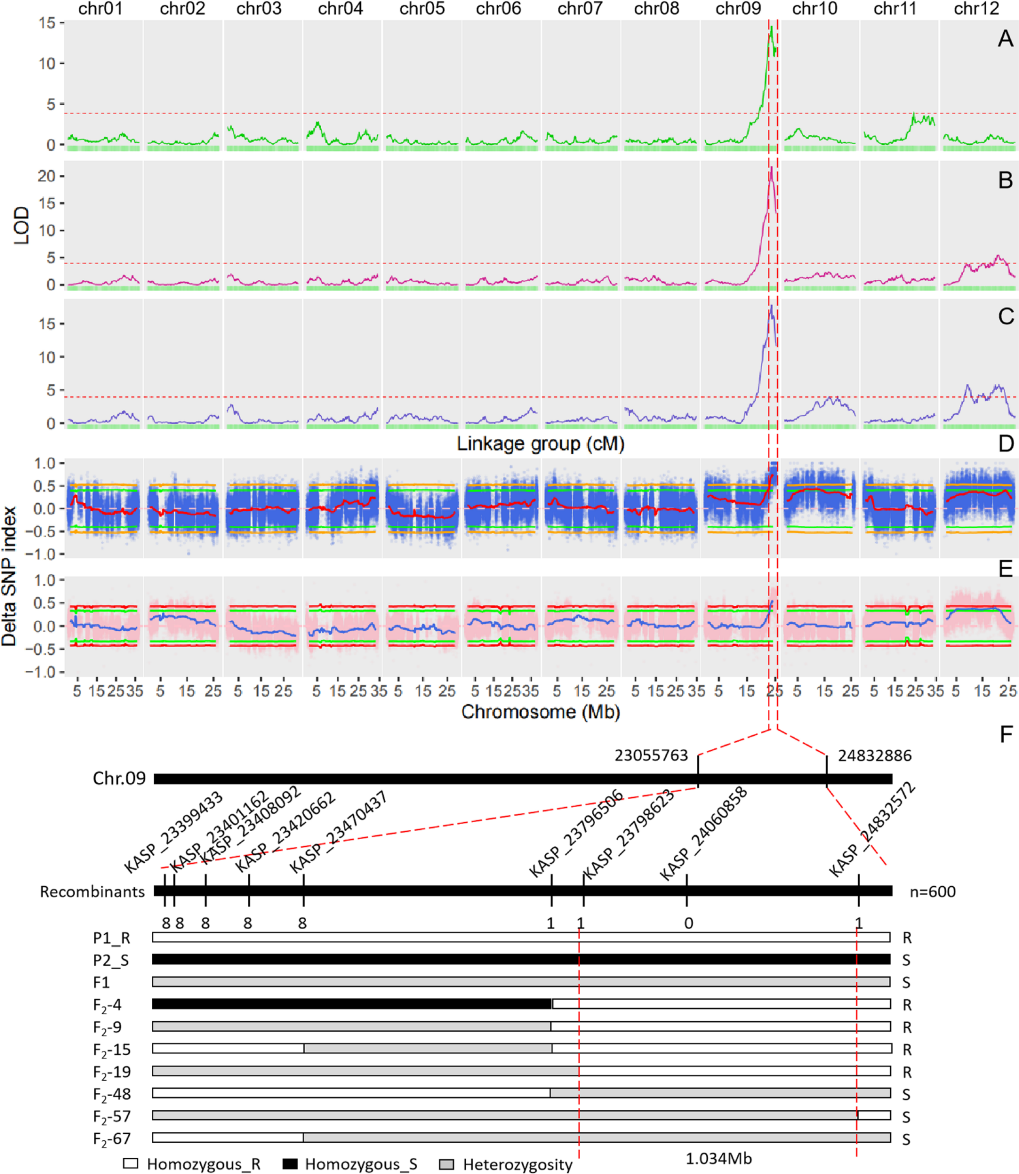
QTL mapping for downy mildew resistance in melon and fine mapping of *DM9.1*. **(A)** QTL mapping of downy mildew resistance at the early growth stage based on the genetic map. **(B)** QTL mapping of downy mildew resistance at the middle growth stage based on the genetic map. **(C)** QTL mapping of downy mildew resistance at the late growth stage based on the genetic map. The dash line represents the cutoff value of LOD at P < 0.05 significant level after 1000 permutation tests. **(D)** QTL mapping of downy mildew resistance in the F2 population of PI 442177 and Huangtu based on QTL-seq analysis. **(E)** QTL mapping of downy mildew resistance in the F2 population of PI 442177 and Huangdanzi based on QTL-seq analysis. **(F)** Fine mapping of *DM9.1* in the F2 population of PI 442177 and Huangdanzi using KASP markers.

The single QTL method provided by R/qtl2 was used to map the QTLs for DM resistance at each growth stage. The significant LOD threshold at p < 0.05 for the inferred QTLs was estimated by 1000 permutation tests. The significant LOD thresholds for the early, middle and late growth stages were 3.86, 3.96 and 3.93, respectively. The significant QTLs were identified for each growth stage (**Table 2**), and their locations are shown in **Figure 3**. At the early growth stage, two QTLs locating on chromosomes of 9 and 11 were detected, which were named as *eDM9.1* and *eDM11.1*, respectively (**Figure 3A**). PVEs of *eDM9.1* and *eDM11.1* were 28.0% and 4.2%, respectively. At the middle growth stage, two QTLs locating on chromosomes of 9 and 12 were identified for DM resistance, which were named as *mDM9.1* and *mDM12.1* (**Figure 3B**). Of which, *mDM9.1* with the PVE of 37.7% exhibited a major effect, while *mDM12.1* with the PVE of 5.2% exhibited a minor effect. Four QTLs, locating at chromosomes of 9, 10, and 12, were detected for DM resistance at the late growth stage and named as *lDM9.1*, *lDM10.1*, *lDM12.1*, and *lDM12.2*, respectively (**Figure 3C**). Of which, *lDM9.1* with the PVE of 24.3% had a major effect, while the other three QTLs with small PVEs exhibited minor effects on DM resistance. No co-locations were observed for the minor-effect QTLs with the PVE less than 10% at different growth stages. However, the major-effect QTLs overlapped at the end of chromosome 9 for all the three growth stages, indicating that it is a QTL conferring whole-stage resistance to DM.

**Table 2:** Summary statistics on the detected QTLs.

Growth stage	QTL	Chromosome	Peak LOD	Peak Marker position (cM)	95% Bayes credible interval (bp)	Gene number	PVE* (%)
Early stage	*eDM9.1*	9	14.6	87.6	23628642-24168798	77	28.0
	*eDM11.1*	11	4.0	61.8	29889003-33998274	537	4.2
Middle stage	*mDM9.1*	9	21.8	87.3	24000068-24083436	9	37.7
	*mDM12.1*	12	5.4	64.0	10949874-25466928	1096	5.2
Late stage	*lDM9.1*	9	17.9	87.3	23628642-24474111	111	24.3
	*lDM10.1*	10	4.0	49.1	3775282-21837102	1037	5.6
	*lDM12.1*	12	5.8	28.0	7238618-15939013	462	2.5
	*lDM12.2*	12	5.8	65.0	22771741-25278793	346	4.3

PVE, Phenotypic variance explained. The symbol "*" represents the annotation of PVE.

### Genetic mapping of DM resistance based on QTL-seq approach

QTL-seq approach was also used to map the QTLs conferring DM resistance. Whole-genome re-sequencing approach was performed for PI 442177, Huangtu, and the two bulks, generating a total of 30.28 G sequencing data. The sequencing depths for the parents and bulks were about 10× and 25×, respectively. A total of 3,112,726 SNPs and 734,564 InDels were identified by GATK4. The variants were further filtered according to the parental genotypes. The filtered SNPs were used to calculate the SNP-index for each bulk and the delta SNP-index between the two bulks. The results are shown in **Figure 3D**. At the significant level of p < 0.05, one QTL was detected at the interval of 22.4-25.2 Mb on chromosome 9 and four QTLs were detected on chromosome 10 (**Table S1**). At the significant level of p < 0.01, only the QTL locating at the interval of 23.1-25.2 Mb on chromosome 9 was detected (**Table S1**).

Whole-genome re-sequencing produced a total of 45.29 G data for Huangdanzi, PI 442177, and the two pools. The sequencing depths were over 7 × and 25 × for the two parents and two pools, respectively. A total of 3,594,752 SNPs and 659,052 InDels were identified. QTL-seq analysis was performed using the filtered variants. At the significant level of p < 0.05, one QTL was detected at the interval of 23.0-25.2 Mb on chromosome 9 and two QTLs were detected on chromosome 12 (**Figure 3E**; **Table S1**). At the significant level of p < 0.01, only the QTL locating at the interval of 23.3-25.2 Mb was detected on chromosome 9 (**Table S1**).

### Fine mapping of *DM9.1* using KASP assay

The QTL locating at the end of chromosome 9 not only exhibited a major effect to DM resistance but also was consistently detected in the two F_2_ populations by both linkage map and QTL-seq analysis. As a result, it was selected for further analysis. To avoid missing the candidate genes, the significant intervals detected by both the linkage map and QTL-seq analysis were merged into one QTL. The merged QTL was named *DM9.1* and delimited at 22.0-25.2 Mb on chromosome 9.

To fine map *DM9.1* into a smaller interval, KASP assay was carried out on the F_2_ population derived from the parental lines of PI 442177 and Huangdanzi, which comprised of 600 individuals. A total of 66 high-quality SNPs in the mapping interval were selected and converted to KASP markers (**Table S2**). KASP assay showed that 33 markers were successfully amplified and exhibited polymorphism between the parental lines of PI 442177 and Huangdanzi. A total of 34 DM-resistant individuals and 51 DM-susceptible individuals in the F_2_ population were selected for fine mapping. The recombinants were identified based on the phenotypes and genotyping results. Finally, *DM9.1* was narrowed down to a 1.0 Mb region flanking by KASP_23798623 and KASP_24832572 (**Figure 3F**).

According to the annotation of reference genome, there were 133 genes in the fine mapping interval. Effects of the variants on the annotated genes were assessed using snpEff. A total of 115 genes were affected by the variants. The IDs and functions of these genes are listed in **Table S3**. Four genes were highly impacted by the variants, including *UDP-glucose 4-epimerase family protein*, *WD repeat-containing protein 44* and two unknown proteins (*MELO3C005854.2*, *MELO3C034011.2*). While, moderate and low impacts were observed on more than 30 genes (**Table S3**). The large number of genes in the mapping interval made it difficult to determine the candidate genes for *DM9.1*.

**Figure 4:**
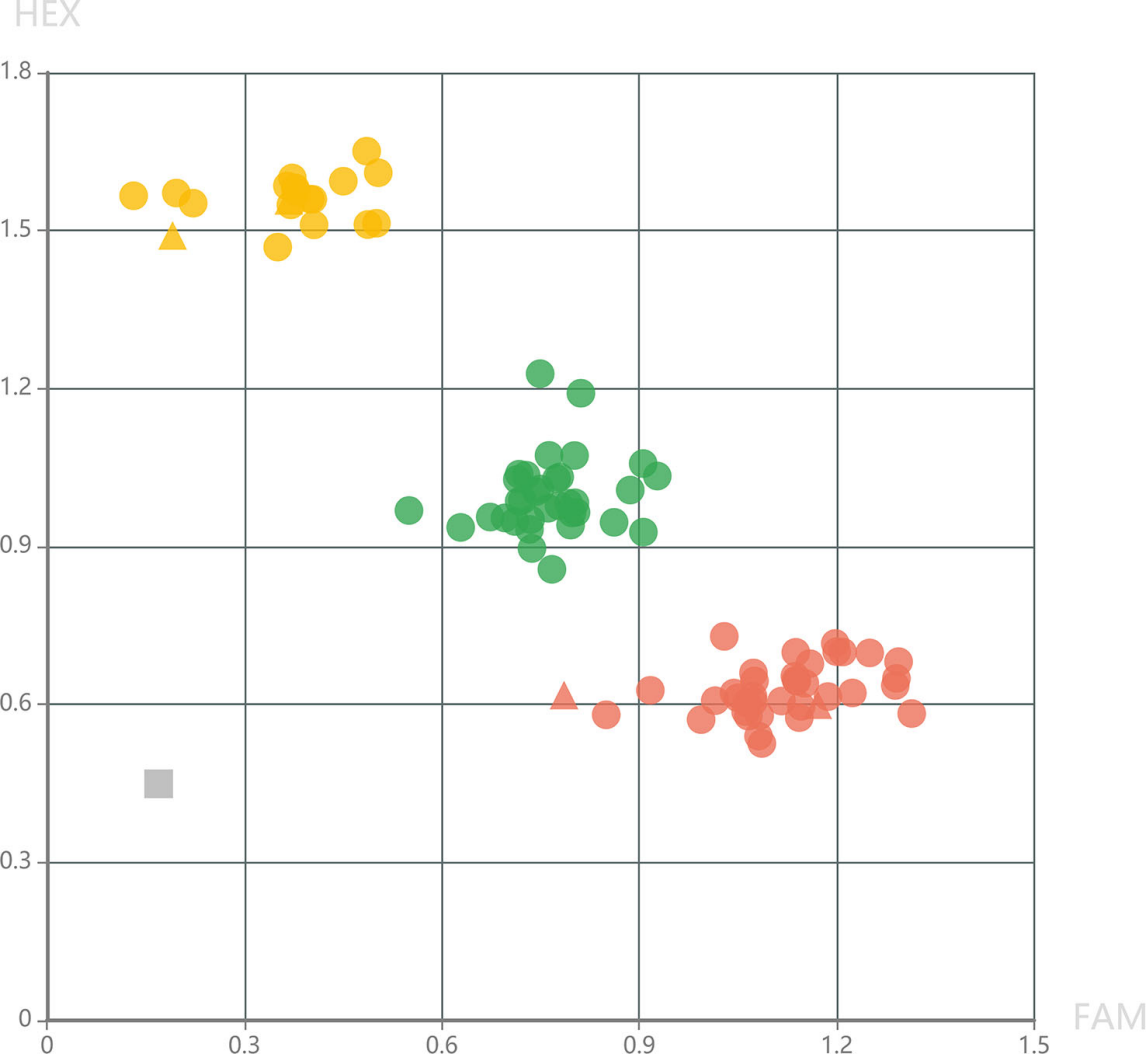
Genotyping pattern of the selected F_2_ individuals derived from the cross of PI 442177 and Huangdanzi using the KASP_24060858 marker. The red cycles represent the homologous resistant genotypes. The yellow cycles represent the homologous susceptible genotypes. The green cycles represent the heterozygous susceptible genotypes. The triangles represent the positive controls of resistant and susceptible genotypes. The gray rectangle represents the negative control (without DNA templates).

To provide high-quality markers for marker-assisted selection of DM resistance, KASP markers were further developed in the fine mapping interval. Genotyping results of KASP_24060858 showed that all the 34 DM-resistant individuals exhibited homologous genotypes, while, the 51 DM-susceptible individuals exhibited homologous or heterozygous genotypes at this locus (**Figure 4**). This result demonstrated that KASP_24060858 was a marker co-segregating with the recessive DM resistance.

## Discussion

DM is a major disease frequently causing great economic loss in melon production. Breeding DM-resistant cultivars is the most efficient approach for disease control. Although several DM-resistant melon accessions had been identified, only a small number of QTLs or genes had been mapped or cloned, which lag far behind the requirements of DM-resistant breeding. To address this problem, the DM-resistant accession PI 442177 was used in this study to map the DM-resistant genes with the aims of providing useful molecular markers and gene resources for melon DM-resistant breeding.

Only one segregating population was used to map the targeted genes in most studies. However, it was reported that the effects of QTLs were prone to be affected by the genetic backgrounds, which means that some QTLs only can be detected under specific background (Paccapelo et al., 2022). In this study, to eliminate the influence of genetic background on QTL mapping, two F_2_ populations were constructed using a DM-resistant parental line and two different DM-susceptible parental lines. This experimental design can significantly improve the accuracy of QTL mapping for DM resistance.

For bi-parental genetic populations, linkage map and QTL-seq are the most frequently used methods to map the targeted genes. Linkage map-based QTL analysis can locate even the minor-effect QTLs and estimate their PVEs, but it is time-consuming (Han et al., 2022). QTL-seq is currently a more popular method to locate the targeted genes because of its high efficiency (Takagi et al., 2013). However, it only can map the major-effect QTLs in most cases. In the previous studies, either linkage map or QTL-seq was used to map targeted genes in melon (Toporek et al., 2021). To get a more comprehensive understanding on the locations of DM-resistant QTLs, both the two methods were used in this study. Overlap of the intervals delimiting by the two methods also validated the accuracy of the mapping results.

At different growth stages, responses of plant to stresses may be determined by different mechanisms and genes, which is called stage-specific tolerance (Dinglasan et al., 2021). For example, different QTLs for salt tolerance in rice were detected at seedling and reproductive stages, respectively (Singh et al., 2021). DM can infect melon at whole growth stage. In this study, to test whether different DM-resistant genes play roles in melon different growth stages, the DM infection levels were measured at early, middle, and late growth stages, respectively. Different minor effect QTLs were detected at different stages. However, the major-effect QTL *DM9.1* was consistently detected at the three stages, demonstrating that *DM9.1* is not only a major-effect QTL but also has function at melon whole growth stage. The major-effect and whole-stage QTL has great value in melon DM-resistant breeding.

Recently, using DM-resistant accession MR-1 and a genetically characterized isolate of *P. cubensis* (clade1, mating type A2), two major-effect QTLs for DM resistance were identified in melon, which located on chromosome 8 and 10, respectively, and *Mildew resistance locus o* (*mlo*) was speculated as the candidate gene for the QTL on chromosome 10 (Toporek et al., 2021). However, in our study, using PI 442177 as the DM-resistant parent line, only a major QTL locating on chromosome 9 was detected. Although a minor QTL was also detected on chromosome 9 (0.9-19.5 Mb for Greenhouse 2, 0.7-1.7 Mb for across all) in MR-1 (Toporek et al., 2021), which was not overlapped with the *DM9.1*. Moreover, the previously reported DM-resistant genes *At1* and *At2* were found to locate on chromosome 2 and 5, respectively (Taler et al., 2004; Toporek et al., 2021). As a result, *DM9.1* is a novel QTL totally different from the previous reports. These results demonstrated that MR-1 and PI 442177 harbored different DM-resistant genes, which can be pyramided in breeding program to further improve DM resistance.

KASP is a high-throughput genotyping assay, which was used to narrow *DM9.1* down into a 1.0 Mb interval on chromosome 9 in this study. The fine mapping interval of *DM9.1* still comprised of 133 genes, which made it difficult to determine the candidate genes using the current genetic populations. However, the mapping interval provided valuable information for further fine map and clone the causal gene of *DM9.1* using genetic populations with larger size in the future. Moreover, the marker KASP_24060858 co-segregating with the recessive DM resistance was developed in this study, which is a powerful genetic tool for maker-assisted selection. To the best of our knowledge, it is the first molecular marker that successfully developed for selection of DM resistance in melon.

Taken together, a stable and major-effect QTL conferring DM resistance was identified in this study by using two F_2_ populations based on linkage map and QTL-seq approaches, and a co-segregating KASP marker was successfully developed. These results will not only provide valuable information for DM-resistant gene cloning, but also offer powerful markers for melon breeding programs with the aim of improving DM resistance.

## Data availability statement

The datasets presented in this study can be found in online repositories. The names of the repository/repositories and accession number(s) can be found below: Genome Sequence Archive (Genomics, Proteomics and Bioinformatics 2021) in National Genomics Data Center (Nucleic Acids Res 2022), China National Center for Bioinformation/Beijing Institute of Genomics, Chinese Academy of Sciences [https://ngdc.cncb.ac.cn/gsa/], CRA009352.

## Author contributions

XZ, HY and QK conceived and designed research. XZ, YL, WY, ML, YY, BL and HY conducted experiments. QK, MW, Y-DG and ZW analyzed data. QK, XZ and Y-DG wrote the manuscript. All authors contributed to the article and approved the submitted version.
