# Supplementary material for: Fine mapping of a novel QTL DM9.1 conferring downy mildew resistance in melon
Source: Front Plant Sci. 2023 Jun 12;14:1202775. doi: 10.3389/fpls.2023.1202775 (PMC10291176; doi:10.3389/fpls.2023.1202775)
Supplement: Supplementary file 2 [file Table_1.docx]

Table S1 The QTLs for downy mildew resistance detected by QTL-seq

| F2 Population | Chr | 95% confidence interval (Mb) | | | 99% confidence interval (Mb) | | |
| --- | --- | --- | --- | --- | --- | --- | --- |
|  |  | Lower limit | Upper limit | Interval | Lower limit | Upper limit | Interval |
| Huangtu × PI442177 | 9 | 22.4 | 25.2 | 2.8 | 23.1 | 25.2 | 2.1 |
|  | 10 | 6.1 | 6.2 | 0.1 |  |  |  |
|  | 10 | 7.3 | 8.0 | 0.7 |  |  |  |
|  | 10 | 8.6 | 9.1 | 0.5 |  |  |  |
|  | 10 | 11.8 | 13.2 | 1.4 |  |  |  |
| Huangdanzi × PI442177 | 9 | 23.0 | 25.2 | 2.2 | 23.3 | 25.2 | 1.9 |
|  | 12 | 10.9 | 13.0 | 2.1 |  |  |  |
|  | 12 | 17.0 | 18.7 | 1.7 |  |  |  |
