# Supplementary material for: Fine mapping of a novel QTL DM9.1 conferring downy mildew resistance in melon
Source: Front Plant Sci. 2023 Jun 12;14:1202775. doi: 10.3389/fpls.2023.1202775 (PMC10291176; doi:10.3389/fpls.2023.1202775)
Supplement: Supplementary file 3 [file Table_3.docx]

**Table S3 Effect of the variants on genes locating at the fine mapping interval**

| Gene ID | Function | variants_impact_HIGH | variants_impact_LOW | variants_impact_MODERATE | variants_impact_MODIFIER | variants_effect_3_prime_UTR_variant | variants_effect_5_prime_UTR_premature_start_codon_gain_variant | variants_effect_5_prime_UTR_variant | variants_effect_conservative_inframe_deletion | variants_effect_conservative_inframe_insertion | variants_effect_disruptive_inframe_deletion | variants_effect_disruptive_inframe_insertion | variants_effect_downstream_gene_variant | variants_effect_frameshift_variant | variants_effect_intron_variant | variants_effect_missense_variant | variants_effect_splice_acceptor_variant | variants_effect_splice_donor_variant | variants_effect_splice_region_variant | variants_effect_start_lost | variants_effect_stop_gained | variants_effect_stop_lost | variants_effect_stop_retained_variant | variants_effect_synonymous_variant | variants_effect_upstream_gene_variant |
| --- | --- | --- | --- | --- | --- | --- | --- | --- | --- | --- | --- | --- | --- | --- | --- | --- | --- | --- | --- | --- | --- | --- | --- | --- | --- |
| MELO3C005767.2 | GDSL esterase/lipase At4g10955 | 0 | 9 | 2 | 97 | 0 | 3 | 14 | 0 | 0 | 0 | 0 | 38 | 0 | 3 | 2 | 0 | 0 | 0 | 0 | 0 | 0 | 0 | 6 | 42 |
| MELO3C005850.2 | S-type anion channel SLAH2 | 0 | 8 | 5 | 45 | 1 | 0 | 0 | 0 | 0 | 0 | 0 | 8 | 0 | 12 | 5 | 0 | 0 | 1 | 0 | 0 | 0 | 0 | 7 | 25 |
| MELO3C005762.2 | AT3g27090/MOJ10_18 | 0 | 6 | 3 | 118 | 5 | 1 | 3 | 0 | 0 | 0 | 0 | 35 | 0 | 0 | 3 | 0 | 0 | 0 | 0 | 0 | 0 | 0 | 5 | 75 |
| MELO3C005800.2 | 50S ribosomal protein L11, chloroplastic | 0 | 6 | 0 | 123 | 0 | 0 | 0 | 0 | 0 | 0 | 0 | 24 | 0 | 47 | 0 | 0 | 0 | 2 | 0 | 0 | 0 | 0 | 4 | 54 |
| MELO3C005839.2 | Glycoside hydrolase family 28 protein/polygalacturonase family protein | 0 | 6 | 0 | 86 | 2 | 0 | 0 | 0 | 0 | 0 | 0 | 62 | 0 | 2 | 0 | 0 | 0 | 0 | 0 | 0 | 0 | 0 | 6 | 20 |
| MELO3C005765.2 | RING/U-box protein | 0 | 5 | 3 | 101 | 2 | 0 | 0 | 0 | 0 | 0 | 0 | 26 | 0 | 25 | 3 | 0 | 0 | 1 | 0 | 0 | 0 | 0 | 4 | 49 |
| MELO3C005855.2 | Topoisomerase 1-associated factor 1 | 0 | 5 | 3 | 127 | 6 | 0 | 0 | 0 | 0 | 0 | 0 | 40 | 0 | 21 | 3 | 0 | 0 | 1 | 0 | 0 | 0 | 0 | 4 | 61 |
| MELO3C005764.2 | Galactose-binding protein isoform 2 | 0 | 5 | 0 | 61 | 0 | 0 | 0 | 0 | 0 | 0 | 0 | 33 | 0 | 1 | 0 | 0 | 0 | 0 | 0 | 0 | 0 | 0 | 5 | 27 |
| MELO3C005861.2 | Hexosyltransferase | 0 | 4 | 2 | 100 | 1 | 1 | 1 | 0 | 0 | 0 | 0 | 37 | 0 | 30 | 2 | 0 | 0 | 1 | 0 | 0 | 0 | 0 | 3 | 31 |
| MELO3C005799.2 | Hexosyltransferase | 0 | 4 | 0 | 134 | 1 | 0 | 3 | 0 | 0 | 0 | 0 | 41 | 0 | 15 | 0 | 0 | 0 | 1 | 0 | 0 | 0 | 0 | 3 | 75 |
| MELO3C033830.2 | Glycosyl transferase, family 31 | 0 | 3 | 6 | 92 | 2 | 0 | 2 | 0 | 0 | 0 | 0 | 47 | 0 | 7 | 6 | 0 | 0 | 0 | 0 | 0 | 0 | 0 | 3 | 34 |
| MELO3C005795.2 | Starch synthase, chloroplastic/amyloplastic | 0 | 3 | 2 | 22 | 8 | 0 | 0 | 0 | 0 | 0 | 0 | 0 | 0 | 11 | 2 | 0 | 0 | 1 | 0 | 0 | 0 | 0 | 2 | 4 |
| MELO3C005857.2 | cytochrome P450 89A2-like | 0 | 3 | 2 | 94 | 0 | 0 | 0 | 0 | 0 | 0 | 0 | 18 | 0 | 2 | 2 | 0 | 0 | 0 | 0 | 0 | 0 | 0 | 3 | 74 |
| MELO3C005777.2 | serine/threonine-protein kinase WNK8-like isoform X1 | 0 | 3 | 1 | 59 | 1 | 0 | 1 | 0 | 0 | 0 | 0 | 8 | 0 | 7 | 1 | 0 | 0 | 0 | 0 | 0 | 0 | 0 | 3 | 42 |
| MELO3C005783.2 | metal-nicotianamine transporter YSL1 | 0 | 3 | 1 | 69 | 12 | 0 | 0 | 0 | 0 | 0 | 0 | 45 | 0 | 12 | 1 | 0 | 0 | 0 | 0 | 0 | 0 | 0 | 3 | 0 |
| MELO3C005792.2 | alcohol dehydrogenase-like 4 | 0 | 3 | 0 | 67 | 0 | 0 | 0 | 0 | 0 | 0 | 0 | 24 | 0 | 41 | 0 | 0 | 0 | 1 | 0 | 0 | 0 | 0 | 2 | 3 |
| MELO3C005860.2 | Dead box ATP-dependent RNA helicase, putative | 0 | 3 | 0 | 81 | 0 | 0 | 0 | 0 | 0 | 0 | 0 | 9 | 0 | 34 | 0 | 0 | 0 | 2 | 0 | 0 | 0 | 0 | 1 | 40 |
| MELO3C005769.2 | UDP-glucose 4-epimerase family protein | 1 | 2 | 3 | 42 | 0 | 0 | 0 | 0 | 0 | 0 | 0 | 6 | 1 | 13 | 3 | 0 | 0 | 0 | 0 | 0 | 0 | 0 | 2 | 23 |
| MELO3C005772.2 | transcription factor WER-like | 0 | 2 | 2 | 16 | 1 | 0 | 0 | 0 | 0 | 0 | 0 | 13 | 0 | 0 | 2 | 0 | 0 | 0 | 0 | 0 | 0 | 0 | 2 | 2 |
| MELO3C005798.2 | StAR-related lipid transfer 7, mitochondrial | 0 | 2 | 2 | 80 | 0 | 0 | 3 | 0 | 0 | 0 | 0 | 25 | 0 | 9 | 2 | 0 | 0 | 1 | 0 | 0 | 0 | 0 | 1 | 44 |
| MELO3C005775.2 | BTB/POZ domain protein | 0 | 2 | 1 | 59 | 4 | 1 | 3 | 0 | 0 | 0 | 0 | 24 | 0 | 8 | 1 | 0 | 0 | 0 | 0 | 0 | 0 | 0 | 1 | 20 |
| MELO3C005748.2 | Endoglucanase | 0 | 2 | 0 | 14 | 0 | 0 | 1 | 0 | 0 | 0 | 0 | 0 | 0 | 6 | 0 | 0 | 0 | 0 | 0 | 0 | 0 | 0 | 2 | 7 |
| MELO3C005757.2 | Luminal binding heat shock protein 70 | 0 | 2 | 0 | 33 | 1 | 0 | 1 | 0 | 0 | 0 | 0 | 6 | 0 | 5 | 0 | 0 | 0 | 0 | 0 | 0 | 0 | 0 | 2 | 20 |
| MELO3C005776.2 | Plant UBX domain-containing protein 10 | 0 | 2 | 0 | 92 | 0 | 1 | 1 | 0 | 0 | 0 | 0 | 43 | 0 | 16 | 0 | 0 | 0 | 0 | 0 | 0 | 0 | 0 | 1 | 32 |
| MELO3C005787.2 | Alpha/beta hydrolase-1 | 0 | 2 | 0 | 65 | 0 | 0 | 1 | 0 | 0 | 0 | 0 | 52 | 0 | 5 | 0 | 0 | 0 | 0 | 0 | 0 | 0 | 0 | 2 | 7 |
| MELO3C005854.2 | Unknown protein | 1 | 1 | 4 | 69 | 0 | 0 | 0 | 0 | 0 | 0 | 0 | 40 | 1 | 0 | 4 | 0 | 0 | 0 | 0 | 0 | 0 | 0 | 1 | 29 |
| MELO3C005791.2 | NAC domain-containing protein 12 | 0 | 1 | 3 | 79 | 1 | 0 | 0 | 1 | 2 | 0 | 0 | 33 | 0 | 2 | 0 | 0 | 0 | 0 | 0 | 0 | 0 | 0 | 1 | 43 |
| MELO3C034011.2 | Unknown protein | 1 | 1 | 3 | 95 | 0 | 0 | 6 | 0 | 0 | 0 | 0 | 51 | 0 | 2 | 3 | 0 | 1 | 1 | 0 | 0 | 0 | 0 | 0 | 38 |
| MELO3C005784.2 | DUF538 family protein | 0 | 1 | 2 | 78 | 0 | 0 | 0 | 0 | 0 | 0 | 0 | 29 | 0 | 0 | 2 | 0 | 0 | 0 | 0 | 0 | 0 | 0 | 1 | 49 |
| MELO3C005761.2 | Auxin response factor | 0 | 1 | 1 | 54 | 1 | 0 | 0 | 0 | 0 | 0 | 0 | 15 | 0 | 9 | 1 | 0 | 0 | 0 | 0 | 0 | 0 | 0 | 1 | 29 |
| MELO3C005788.2 | GDSL esterase/lipase At5g41890 | 0 | 1 | 1 | 112 | 0 | 0 | 0 | 0 | 0 | 0 | 0 | 44 | 0 | 18 | 1 | 0 | 0 | 0 | 0 | 0 | 0 | 0 | 1 | 50 |
| MELO3C005834.2 | Scarecrow-like protein 8, putative | 0 | 1 | 1 | 23 | 1 | 0 | 0 | 0 | 0 | 0 | 0 | 10 | 0 | 0 | 1 | 0 | 0 | 0 | 0 | 0 | 0 | 0 | 1 | 12 |
| MELO3C033831.2 | Cytochrome b561 and DOMON domain-containing protein | 0 | 1 | 1 | 78 | 0 | 0 | 0 | 0 | 0 | 0 | 0 | 50 | 0 | 0 | 1 | 0 | 0 | 0 | 0 | 0 | 0 | 0 | 1 | 28 |
| MELO3C005751.2 | Trehalase | 0 | 1 | 0 | 54 | 1 | 0 | 0 | 0 | 0 | 0 | 0 | 35 | 0 | 0 | 0 | 0 | 0 | 0 | 0 | 0 | 0 | 0 | 1 | 18 |
| MELO3C005759.2 | 30S ribosomal protein S6 alpha, chloroplastic | 0 | 1 | 0 | 74 | 0 | 0 | 0 | 0 | 0 | 0 | 0 | 16 | 0 | 5 | 0 | 0 | 0 | 0 | 0 | 0 | 0 | 0 | 1 | 53 |
| MELO3C005785.2 | Embryogenesis-associated protein EMB8 | 0 | 1 | 0 | 87 | 0 | 0 | 0 | 0 | 0 | 0 | 0 | 18 | 0 | 11 | 0 | 0 | 0 | 0 | 0 | 0 | 0 | 0 | 1 | 58 |
| MELO3C005796.2 | Avr9/Cf-9 rapidly elicited protein | 0 | 1 | 0 | 39 | 1 | 0 | 0 | 0 | 0 | 0 | 0 | 34 | 0 | 0 | 0 | 0 | 0 | 0 | 0 | 0 | 0 | 0 | 1 | 4 |
| MELO3C005805.2 | heavy metal-associated isoprenylated plant protein 21-like | 0 | 1 | 0 | 14 | 0 | 0 | 0 | 0 | 0 | 0 | 0 | 4 | 0 | 4 | 0 | 0 | 0 | 1 | 0 | 0 | 0 | 0 | 0 | 7 |
| MELO3C005816.2 | Receptor protein kinase, putative | 0 | 1 | 0 | 5 | 0 | 0 | 0 | 0 | 0 | 0 | 0 | 4 | 0 | 1 | 0 | 0 | 0 | 0 | 0 | 0 | 0 | 0 | 1 | 0 |
| MELO3C005824.2 | Protein DYAD | 0 | 1 | 0 | 1 | 0 | 0 | 0 | 0 | 0 | 0 | 0 | 1 | 0 | 1 | 0 | 0 | 0 | 1 | 0 | 0 | 0 | 0 | 0 | 0 |
| MELO3C005841.2 | Metal ion binding protein | 0 | 1 | 0 | 120 | 0 | 0 | 0 | 0 | 0 | 0 | 0 | 16 | 0 | 6 | 0 | 0 | 0 | 0 | 0 | 0 | 0 | 0 | 1 | 98 |
| MELO3C005847.2 | Membrane-associated kinase regulator, putative | 0 | 1 | 0 | 0 | 0 | 0 | 0 | 0 | 0 | 0 | 0 | 0 | 0 | 0 | 0 | 0 | 0 | 0 | 0 | 0 | 0 | 0 | 1 | 0 |
| MELO3C005840.2 | protein REVEILLE 6-like | 0 | 0 | 4 | 175 | 1 | 0 | 0 | 0 | 0 | 0 | 0 | 57 | 0 | 19 | 4 | 0 | 0 | 0 | 0 | 0 | 0 | 0 | 0 | 98 |
| MELO3C005763.2 | Glutaredoxin | 0 | 0 | 3 | 103 | 3 | 0 | 2 | 0 | 0 | 0 | 0 | 75 | 0 | 0 | 3 | 0 | 0 | 0 | 0 | 0 | 0 | 0 | 0 | 23 |
| MELO3C005797.2 | Unknown protein | 0 | 0 | 2 | 56 | 0 | 0 | 0 | 0 | 0 | 0 | 0 | 36 | 0 | 0 | 2 | 0 | 0 | 0 | 0 | 0 | 0 | 0 | 0 | 20 |
| MELO3C005755.2 | ABC transporter F family-like protein | 0 | 0 | 1 | 0 | 0 | 0 | 0 | 0 | 0 | 0 | 0 | 0 | 0 | 0 | 1 | 0 | 0 | 0 | 0 | 0 | 0 | 0 | 0 | 0 |
| MELO3C005789.2 | Protein BRANCHLESS TRICHOME | 0 | 0 | 1 | 55 | 1 | 0 | 0 | 0 | 0 | 0 | 0 | 8 | 0 | 4 | 1 | 0 | 0 | 0 | 0 | 0 | 0 | 0 | 0 | 42 |
| MELO3C005812.2 | ARM repeat superfamily protein | 0 | 0 | 1 | 27 | 1 | 0 | 0 | 0 | 0 | 0 | 0 | 4 | 0 | 6 | 1 | 0 | 0 | 0 | 0 | 0 | 0 | 0 | 0 | 16 |
| MELO3C005815.2 | Trihelix transcription factor GT-2 | 0 | 0 | 1 | 1 | 0 | 0 | 0 | 0 | 0 | 0 | 0 | 0 | 0 | 0 | 1 | 0 | 0 | 0 | 0 | 0 | 0 | 0 | 0 | 1 |
| MELO3C005832.2 | cucumisin-like isoform X1 | 0 | 0 | 1 | 1 | 0 | 0 | 0 | 0 | 0 | 0 | 0 | 1 | 0 | 0 | 1 | 0 | 0 | 0 | 0 | 0 | 0 | 0 | 0 | 0 |
| MELO3C005732.2 | LRR receptor-like kinase | 0 | 0 | 0 | 1 | 0 | 0 | 0 | 0 | 0 | 0 | 0 | 0 | 0 | 0 | 0 | 0 | 0 | 0 | 0 | 0 | 0 | 0 | 0 | 1 |
| MELO3C005734.2 | ethylene-responsive transcription factor CRF2-like | 0 | 0 | 0 | 9 | 0 | 0 | 0 | 0 | 0 | 0 | 0 | 5 | 0 | 0 | 0 | 0 | 0 | 0 | 0 | 0 | 0 | 0 | 0 | 4 |
| MELO3C005735.2 | Cox19-like CHCH family protein | 0 | 0 | 0 | 6 | 0 | 0 | 0 | 0 | 0 | 0 | 0 | 1 | 0 | 0 | 0 | 0 | 0 | 0 | 0 | 0 | 0 | 0 | 0 | 5 |
| MELO3C005736.2 | Cytoplasmic tRNA 2-thiolation protein | 0 | 0 | 0 | 1 | 1 | 0 | 0 | 0 | 0 | 0 | 0 | 0 | 0 | 0 | 0 | 0 | 0 | 0 | 0 | 0 | 0 | 0 | 0 | 0 |
| MELO3C005737.2 | Kinase family protein | 0 | 0 | 0 | 1 | 0 | 0 | 0 | 0 | 0 | 0 | 0 | 0 | 0 | 0 | 0 | 0 | 0 | 0 | 0 | 0 | 0 | 0 | 0 | 1 |
| MELO3C005746.2 | Pectin lyase-like superfamily protein | 0 | 0 | 0 | 1 | 0 | 0 | 0 | 0 | 0 | 0 | 0 | 0 | 0 | 0 | 0 | 0 | 0 | 0 | 0 | 0 | 0 | 0 | 0 | 1 |
| MELO3C005747.2 | ethylene-responsive transcription factor ERF061-like | 0 | 0 | 0 | 1 | 0 | 0 | 0 | 0 | 0 | 0 | 0 | 0 | 0 | 0 | 0 | 0 | 0 | 0 | 0 | 0 | 0 | 0 | 0 | 1 |
| MELO3C005749.2 | long chain acyl-CoA synthetase 4-like | 0 | 0 | 0 | 9 | 0 | 0 | 0 | 0 | 0 | 0 | 0 | 9 | 0 | 0 | 0 | 0 | 0 | 0 | 0 | 0 | 0 | 0 | 0 | 0 |
| MELO3C005752.2 | Short-chain dehydrogenase TIC 32, chloroplastic | 0 | 0 | 0 | 1 | 0 | 0 | 0 | 0 | 0 | 0 | 0 | 0 | 0 | 1 | 0 | 0 | 0 | 0 | 0 | 0 | 0 | 0 | 0 | 0 |
| MELO3C005753.2 | Phosphoribulokinase/uridine kinase | 0 | 0 | 0 | 1 | 0 | 0 | 0 | 0 | 0 | 0 | 0 | 0 | 0 | 0 | 0 | 0 | 0 | 0 | 0 | 0 | 0 | 0 | 0 | 1 |
| MELO3C005756.2 | Glutamine-dependent NAD(+) synthetase | 0 | 0 | 0 | 24 | 1 | 0 | 0 | 0 | 0 | 0 | 0 | 22 | 0 | 0 | 0 | 0 | 0 | 0 | 0 | 0 | 0 | 0 | 0 | 1 |
| MELO3C005758.2 | Bidirectional sugar transporter SWEET | 0 | 0 | 0 | 81 | 1 | 0 | 0 | 0 | 0 | 0 | 0 | 44 | 0 | 0 | 0 | 0 | 0 | 0 | 0 | 0 | 0 | 0 | 0 | 36 |
| MELO3C005760.2 | RING-H2 zinc finger protein RHA4a | 0 | 0 | 0 | 31 | 1 | 0 | 4 | 0 | 0 | 0 | 0 | 11 | 0 | 1 | 0 | 0 | 0 | 0 | 0 | 0 | 0 | 0 | 0 | 14 |
| MELO3C005770.2 | NDK domain-containing protein | 0 | 0 | 0 | 36 | 1 | 0 | 0 | 0 | 0 | 0 | 0 | 21 | 0 | 1 | 0 | 0 | 0 | 0 | 0 | 0 | 0 | 0 | 0 | 13 |
| MELO3C005771.2 | methyltransferase-like protein 17, mitochondrial | 0 | 0 | 0 | 33 | 0 | 0 | 0 | 0 | 0 | 0 | 0 | 12 | 0 | 9 | 0 | 0 | 0 | 0 | 0 | 0 | 0 | 0 | 0 | 12 |
| MELO3C005773.2 | WD repeat-containing protein 44 | 1 | 0 | 0 | 14 | 0 | 0 | 0 | 0 | 0 | 0 | 0 | 13 | 0 | 1 | 0 | 1 | 0 | 0 | 0 | 0 | 0 | 0 | 0 | 1 |
| MELO3C005774.2 | dof zinc finger protein DOF4.6-like | 0 | 0 | 0 | 39 | 4 | 0 | 0 | 0 | 0 | 0 | 0 | 33 | 0 | 0 | 0 | 0 | 0 | 0 | 0 | 0 | 0 | 0 | 0 | 2 |
| MELO3C005778.2 | Oligopeptide transporter, putative | 0 | 0 | 0 | 20 | 0 | 0 | 0 | 0 | 0 | 0 | 0 | 4 | 0 | 1 | 0 | 0 | 0 | 0 | 0 | 0 | 0 | 0 | 0 | 15 |
| MELO3C005779.2 | Metal-dependent protein hydrolase | 0 | 0 | 0 | 7 | 1 | 0 | 0 | 0 | 0 | 0 | 0 | 1 | 0 | 1 | 0 | 0 | 0 | 0 | 0 | 0 | 0 | 0 | 0 | 4 |
| MELO3C005780.2 | Zinc finger matrin-type protein 1, putative isoform 1 | 0 | 0 | 0 | 11 | 0 | 0 | 0 | 0 | 0 | 0 | 0 | 4 | 0 | 0 | 0 | 0 | 0 | 0 | 0 | 0 | 0 | 0 | 0 | 7 |
| MELO3C005781.2 | phosphoribosylformylglycinamidine cyclo-ligase, chloroplastic/mitochondrial | 0 | 0 | 0 | 13 | 1 | 0 | 0 | 0 | 0 | 0 | 0 | 4 | 0 | 4 | 0 | 0 | 0 | 0 | 0 | 0 | 0 | 0 | 0 | 4 |
| MELO3C005782.2 | Erythronate-4-phosphate dehydrogenase family protein, putative | 0 | 0 | 0 | 11 | 0 | 0 | 0 | 0 | 0 | 0 | 0 | 7 | 0 | 0 | 0 | 0 | 0 | 0 | 0 | 0 | 0 | 0 | 0 | 4 |
| MELO3C005786.2 | Cystathionine gamma-synthase | 0 | 0 | 0 | 27 | 0 | 0 | 1 | 0 | 0 | 0 | 0 | 10 | 0 | 0 | 0 | 0 | 0 | 0 | 0 | 0 | 0 | 0 | 0 | 16 |
| MELO3C005790.2 | Electron transporter | 0 | 0 | 0 | 22 | 0 | 0 | 0 | 0 | 0 | 0 | 0 | 11 | 0 | 0 | 0 | 0 | 0 | 0 | 0 | 0 | 0 | 0 | 0 | 11 |
| MELO3C005793.2 | ubiquitin carboxyl-terminal hydrolase 9 | 0 | 0 | 0 | 56 | 0 | 0 | 0 | 0 | 0 | 0 | 0 | 0 | 0 | 0 | 0 | 0 | 0 | 0 | 0 | 0 | 0 | 0 | 0 | 56 |
| MELO3C005794.2 | Glucan endo-1,3-beta-glucosidase, putative | 0 | 0 | 0 | 20 | 0 | 0 | 0 | 0 | 0 | 0 | 0 | 0 | 0 | 0 | 0 | 0 | 0 | 0 | 0 | 0 | 0 | 0 | 0 | 20 |
| MELO3C005801.2 | 1-aminocyclopropane-1-carboxylate oxidase, putative | 0 | 0 | 0 | 106 | 0 | 0 | 0 | 0 | 0 | 0 | 0 | 69 | 0 | 0 | 0 | 0 | 0 | 0 | 0 | 0 | 0 | 0 | 0 | 37 |
| MELO3C005802.2 | Mitochondrial import inner membrane translocase subunit Tim13 | 0 | 0 | 0 | 88 | 0 | 0 | 0 | 0 | 0 | 0 | 0 | 48 | 0 | 0 | 0 | 0 | 0 | 0 | 0 | 0 | 0 | 0 | 0 | 40 |
| MELO3C005803.2 | WAT1-related protein | 0 | 0 | 0 | 14 | 0 | 0 | 0 | 0 | 0 | 0 | 0 | 4 | 0 | 3 | 0 | 0 | 0 | 0 | 0 | 0 | 0 | 0 | 0 | 7 |
| MELO3C005804.2 | E3 ubiquitin-protein ligase MARCH9 isoform X3 | 0 | 0 | 0 | 16 | 0 | 0 | 0 | 0 | 0 | 0 | 0 | 9 | 0 | 1 | 0 | 0 | 0 | 0 | 0 | 0 | 0 | 0 | 0 | 6 |
| MELO3C005806.2 | alpha-L-fucosidase 1-like | 0 | 0 | 0 | 25 | 0 | 0 | 1 | 0 | 0 | 0 | 0 | 9 | 0 | 2 | 0 | 0 | 0 | 0 | 0 | 0 | 0 | 0 | 0 | 13 |
| MELO3C005807.2 | B3 domain-containing transcription factor NGA1-like | 0 | 0 | 0 | 16 | 0 | 0 | 0 | 0 | 0 | 0 | 0 | 8 | 0 | 0 | 0 | 0 | 0 | 0 | 0 | 0 | 0 | 0 | 0 | 8 |
| MELO3C005809.2 | Cinnamyl alcohol dehydrogenase-like protein | 0 | 0 | 0 | 10 | 0 | 0 | 0 | 0 | 0 | 0 | 0 | 7 | 0 | 3 | 0 | 0 | 0 | 0 | 0 | 0 | 0 | 0 | 0 | 0 |
| MELO3C005810.2 | At1g73940/F2P9_19 | 0 | 0 | 0 | 14 | 0 | 0 | 0 | 0 | 0 | 0 | 0 | 0 | 0 | 0 | 0 | 0 | 0 | 0 | 0 | 0 | 0 | 0 | 0 | 14 |
| MELO3C005811.2 | RING-H2 finger protein ATL79-like | 0 | 0 | 0 | 18 | 0 | 0 | 0 | 0 | 0 | 0 | 0 | 4 | 0 | 0 | 0 | 0 | 0 | 0 | 0 | 0 | 0 | 0 | 0 | 14 |
| MELO3C005813.2 | Dehydration-responsive family protein | 0 | 0 | 0 | 10 | 1 | 0 | 0 | 0 | 0 | 0 | 0 | 5 | 0 | 0 | 0 | 0 | 0 | 0 | 0 | 0 | 0 | 0 | 0 | 4 |
| MELO3C005814.2 | transmembrane protein 120 homolog | 0 | 0 | 0 | 10 | 0 | 0 | 0 | 0 | 0 | 0 | 0 | 4 | 0 | 0 | 0 | 0 | 0 | 0 | 0 | 0 | 0 | 0 | 0 | 6 |
| MELO3C005817.2 | Aquaporin-like protein | 0 | 0 | 0 | 6 | 0 | 0 | 1 | 0 | 0 | 0 | 0 | 3 | 0 | 1 | 0 | 0 | 0 | 0 | 0 | 0 | 0 | 0 | 0 | 1 |
| MELO3C005818.2 | Aquaporin-like protein | 0 | 0 | 0 | 3 | 0 | 0 | 0 | 0 | 0 | 0 | 0 | 3 | 0 | 0 | 0 | 0 | 0 | 0 | 0 | 0 | 0 | 0 | 0 | 0 |
| MELO3C005819.2 | E3 ubiquitin-protein ligase KEG | 0 | 0 | 0 | 1 | 0 | 0 | 0 | 0 | 0 | 0 | 0 | 0 | 0 | 0 | 0 | 0 | 0 | 0 | 0 | 0 | 0 | 0 | 0 | 1 |
| MELO3C005820.2 | Ulp1 peptidase-like | 0 | 0 | 0 | 2 | 0 | 0 | 0 | 0 | 0 | 0 | 0 | 1 | 0 | 0 | 0 | 0 | 0 | 0 | 0 | 0 | 0 | 0 | 0 | 1 |
| MELO3C005821.2 | mRNA, clone: RTFL01-46-D12 | 0 | 0 | 0 | 2 | 0 | 0 | 0 | 0 | 0 | 0 | 0 | 1 | 0 | 0 | 0 | 0 | 0 | 0 | 0 | 0 | 0 | 0 | 0 | 1 |
| MELO3C005822.2 | PLAT domain-containing protein 1 | 0 | 0 | 0 | 1 | 0 | 0 | 0 | 0 | 0 | 0 | 0 | 1 | 0 | 0 | 0 | 0 | 0 | 0 | 0 | 0 | 0 | 0 | 0 | 0 |
| MELO3C005823.2 | 8-amino-7-oxononanoate synthase | 0 | 0 | 0 | 2 | 0 | 0 | 0 | 0 | 0 | 0 | 0 | 1 | 0 | 0 | 0 | 0 | 0 | 0 | 0 | 0 | 0 | 0 | 0 | 1 |
| MELO3C005826.2 | Short-chain dehydrogenase TIC 32, chloroplastic | 0 | 0 | 0 | 1 | 0 | 0 | 0 | 0 | 0 | 0 | 0 | 0 | 0 | 0 | 0 | 0 | 0 | 0 | 0 | 0 | 0 | 0 | 0 | 1 |
| MELO3C005827.2 | Short-chain dehydrogenase TIC 32, chloroplastic | 0 | 0 | 0 | 8 | 1 | 0 | 0 | 0 | 0 | 0 | 0 | 0 | 0 | 0 | 0 | 0 | 0 | 0 | 0 | 0 | 0 | 0 | 0 | 7 |
| MELO3C005828.2 | SKP1-like protein 21 | 0 | 0 | 0 | 8 | 0 | 0 | 0 | 0 | 0 | 0 | 0 | 1 | 0 | 0 | 0 | 0 | 0 | 0 | 0 | 0 | 0 | 0 | 0 | 7 |
| MELO3C005829.2 | Plant/K24M7-17 protein | 0 | 0 | 0 | 1 | 1 | 0 | 0 | 0 | 0 | 0 | 0 | 0 | 0 | 0 | 0 | 0 | 0 | 0 | 0 | 0 | 0 | 0 | 0 | 0 |
| MELO3C005830.2 | Ribosomal protein S9 | 0 | 0 | 0 | 2 | 0 | 0 | 0 | 0 | 0 | 0 | 0 | 0 | 0 | 1 | 0 | 0 | 0 | 0 | 0 | 0 | 0 | 0 | 0 | 1 |
| MELO3C005831.2 | Protein DETOXIFICATION | 0 | 0 | 0 | 3 | 0 | 0 | 0 | 0 | 0 | 0 | 0 | 1 | 0 | 0 | 0 | 0 | 0 | 0 | 0 | 0 | 0 | 0 | 0 | 2 |
| MELO3C005837.2 | RNA-binding protein-like RNA recognition motif protein | 0 | 0 | 0 | 4 | 0 | 0 | 0 | 0 | 0 | 0 | 0 | 0 | 0 | 0 | 0 | 0 | 0 | 0 | 0 | 0 | 0 | 0 | 0 | 4 |
| MELO3C005838.2 | Site-determining protein | 0 | 0 | 0 | 28 | 0 | 0 | 0 | 0 | 0 | 0 | 0 | 28 | 0 | 0 | 0 | 0 | 0 | 0 | 0 | 0 | 0 | 0 | 0 | 0 |
| MELO3C005842.2 | U1 small nuclear ribonucleoprotein component SNU71 | 0 | 0 | 0 | 11 | 0 | 0 | 0 | 0 | 0 | 0 | 0 | 1 | 0 | 0 | 0 | 0 | 0 | 0 | 0 | 0 | 0 | 0 | 0 | 10 |
| MELO3C005843.2 | WRKY transcription factor | 0 | 0 | 0 | 2 | 0 | 0 | 0 | 0 | 0 | 0 | 0 | 1 | 0 | 0 | 0 | 0 | 0 | 0 | 0 | 0 | 0 | 0 | 0 | 1 |
| MELO3C005844.2 | 60S ribosomal protein l9 | 0 | 0 | 0 | 1 | 0 | 0 | 0 | 0 | 0 | 0 | 0 | 1 | 0 | 0 | 0 | 0 | 0 | 0 | 0 | 0 | 0 | 0 | 0 | 0 |
| MELO3C005845.2 | trafficking protein particle complex subunit 1 | 0 | 0 | 0 | 2 | 0 | 0 | 0 | 0 | 0 | 0 | 0 | 1 | 0 | 0 | 0 | 0 | 0 | 0 | 0 | 0 | 0 | 0 | 0 | 1 |
| MELO3C005846.2 | Unknown protein | 0 | 0 | 0 | 1 | 0 | 0 | 0 | 0 | 0 | 0 | 0 | 1 | 0 | 0 | 0 | 0 | 0 | 0 | 0 | 0 | 0 | 0 | 0 | 0 |
| MELO3C005849.2 | p-loop containing nucleoside triphosphate hydrolases superfamily protein | 0 | 0 | 0 | 31 | 0 | 0 | 0 | 0 | 0 | 0 | 0 | 0 | 0 | 0 | 0 | 0 | 0 | 0 | 0 | 0 | 0 | 0 | 0 | 31 |
| MELO3C005851.2 | Membrane-associated kinase regulator-like protein, putative | 0 | 0 | 0 | 24 | 0 | 0 | 0 | 0 | 0 | 0 | 0 | 24 | 0 | 0 | 0 | 0 | 0 | 0 | 0 | 0 | 0 | 0 | 0 | 0 |
| MELO3C005853.2 | Unknown protein | 0 | 0 | 0 | 26 | 0 | 0 | 0 | 0 | 0 | 0 | 0 | 26 | 0 | 0 | 0 | 0 | 0 | 0 | 0 | 0 | 0 | 0 | 0 | 0 |
| MELO3C005858.2 | acidic endochitinase | 0 | 0 | 0 | 50 | 0 | 0 | 0 | 0 | 0 | 0 | 0 | 9 | 0 | 0 | 0 | 0 | 0 | 0 | 0 | 0 | 0 | 0 | 0 | 41 |
| MELO3C005859.2 | acidic endochitinase-like | 0 | 0 | 0 | 42 | 0 | 0 | 0 | 0 | 0 | 0 | 0 | 14 | 0 | 0 | 0 | 0 | 0 | 0 | 0 | 0 | 0 | 0 | 0 | 28 |
| MELO3C034006.2 | Arabinogalactan peptide-like protein | 0 | 0 | 0 | 5 | 0 | 0 | 0 | 0 | 0 | 0 | 0 | 4 | 0 | 0 | 0 | 0 | 0 | 0 | 0 | 0 | 0 | 0 | 0 | 1 |
| MELO3C034008.2 | Unknown protein | 0 | 0 | 0 | 1 | 0 | 0 | 0 | 0 | 0 | 0 | 0 | 1 | 0 | 0 | 0 | 0 | 0 | 0 | 0 | 0 | 0 | 0 | 0 | 0 |
| MELO3C034009.2 | Cytochrome b561 and domon domain-containing protein | 0 | 0 | 0 | 76 | 0 | 0 | 0 | 0 | 0 | 0 | 0 | 50 | 0 | 1 | 0 | 0 | 0 | 0 | 0 | 0 | 0 | 0 | 0 | 25 |
